# Precision Therapeutics in Pancreatic Cancer: Emerging Targeted, Immune, and Antibody–Drug Conjugate Strategies Exemplified by Adagrasib, Dostarlimab, and Trastuzumab Deruxtecan

**DOI:** 10.3390/jcm15145521

**Published:** 2026-07-14

**Authors:** Piotr Kawczak, Katarzyna Kawczak, Tomasz Bączek

**Affiliations:** 1 Department of Pharmaceutical Chemistry, Faculty of Pharmacy, Medical University of Gdańsk, 80-416 Gdansk, Poland; tomasz.baczek@gumed.edu.pl; 2 PwC Advisory sp. z o.o., sp.k., Aleja Grunwaldzka 472C, 80-309 Gdansk, Poland; 3 Department of Nursing and Medical Rescue, Institute of Health Sciences, Pomeranian University in Słupsk, 76-200 Slupsk, Poland

**Keywords:** pancreatic cancer, molecular profiling, targeted therapy, biomarker-driven therapy, antibody–drug conjugates, KRAS G12C inhibition, PD-1 blockade, HER2, adagrasib, dostarlimab, trastuzumab deruxtecan, precision medicine

## Abstract

Pancreatic cancer remains one of the most aggressive and lethal malignancies, characterized by late-stage diagnosis, profound molecular heterogeneity, and limited responsiveness to conventional cytotoxic therapies. Recent advances in molecular diagnostics and biomarker-driven treatment stratification have accelerated the development of precision therapeutic approaches aimed at improving outcomes in selected patient populations. This review highlights three mechanistically distinct yet complementary therapeutic strategies that illustrate the evolving landscape of personalized pancreatic cancer management. Adagrasib represents targeted inhibition of oncogenic KRAS G12C signaling, reflecting recent progress in directly targeting historically “undruggable” driver mutations. Dostarlimab illustrates the tissue-agnostic application of immune checkpoint blockade in pancreatic cancers harboring mismatch repair deficiency (dMMR) or high microsatellite instability (MSI-H), highlighting the growing importance of biomarker-defined immunotherapy-responsive subsets despite the limited pancreatic cancer-specific clinical evidence currently available. Trastuzumab deruxtecan represents a next-generation HER2-directed antibody–drug conjugate (ADC) and demonstrates the potential of HER2-targeted therapy in the small subgroup of patients with HER2-positive pancreatic cancer, although the available evidence is derived primarily from basket trials and tumor-agnostic clinical development. Collectively, these therapeutic approaches underscore the expanding role of biomarker-guided treatment strategies integrating targeted inhibition, immunotherapy, and precision cytotoxic payload delivery. This review summarizes the molecular rationale, available clinical evidence, therapeutic limitations, and resistance mechanisms associated with these approaches while discussing emerging directions in translational research, rational combination strategies, liquid biopsy applications, and precision oncology that may further refine individualized treatment algorithms for pancreatic cancer.

## 1. Introduction

Pancreatic cancer remains one of the most aggressive and therapeutically challenging malignancies worldwide, with pancreatic ductal adenocarcinoma (PDAC) accounting for approximately 90% of all pancreatic neoplasms [1,2,3]. Despite advances in systemic therapy, PDAC continues to be associated with poor long-term survival, high metastatic potential, rapid disease progression, and profound resistance to conventional cytotoxic treatment [1,2,3,4,5]. The incidence and mortality of pancreatic cancer continue to increase globally, and the disease is projected to become one of the leading causes of cancer-related death in many developed countries over the coming decades [2,4,6]. Because early clinical symptoms are nonspecific and effective population screening strategies remain unavailable, most patients present with unresectable or metastatic disease, making systemic therapy the principal treatment modality [1,3,5,7,8].

Historically, treatment relied predominantly on cytotoxic chemotherapy, including gemcitabine-based regimens and FOLFIRINOX [8,9,10]. Although these approaches improved outcomes in selected patients, their efficacy remains limited by marked molecular heterogeneity and the rapid development of therapeutic resistance [11,12,13]. Increasing the understanding of PDAC biology has therefore driven a transition toward biomarker-guided precision oncology, supported by advances in genomic profiling, molecular pathology, and next-generation sequencing technologies [14,15,16,17,18,19,20,21].

Comprehensive molecular characterization has identified recurrent alterations involving KRAS, TP53, CDKN2A, and SMAD4, together with less common but clinically actionable abnormalities affecting DNA damage repair pathways, HER2 amplification, dMMR, and selected oncogenic KRAS variants [22,23,24,25,26,27,28,29,30]. These discoveries have established the basis for molecular stratification and personalized therapeutic intervention in biologically selected patient populations, while additional targets—including homologous recombination deficiency, NTRK fusions, RET rearrangements, and Claudin 18.2 expression—continue to expand the therapeutic landscape [18,22,23,24,25,26,27,28,29,30].

Among recent advances, direct targeting of KRAS represents a major milestone in PDAC precision medicine. Although KRAS mutations occur in approximately 90% of PDACs, the therapeutically targetable KRAS G12C subtype is present in only a small proportion of patients [23,31,32,33,34,35,36,37,38]. Adagrasib, a selective covalent KRAS G12C inhibitor, has demonstrated encouraging clinical activity in molecularly selected patients with previously treated PDAC and currently represents the strongest pancreatic cancer-specific clinical evidence among the three therapeutic approaches discussed in this review [33,34,35,36,37,38].

Immunotherapy has transformed the management of several malignancies; however, its role in PDAC remains limited because most tumors exhibit low immunogenicity and resistance to immune checkpoint blockade [12,13,39]. Clinical benefit is largely restricted to the rare subgroup of patients with dMMR/MSI-H tumors [40,41,42,43,44,45,46]. Dostarlimab has received tumor-agnostic regulatory approval for dMMR malignancies based primarily on basket trials enrolling multiple solid tumors [41,42,43,44]. Importantly, the currently available PDAC-specific clinical evidence remains limited to small pancreatic cancer subgroups, and its application in PDAC is therefore supported predominantly by biomarker-driven tissue-agnostic evidence rather than pancreatic cancer-specific trials [41,42,43,44].

Similarly, ADCs have emerged as an important therapeutic platform across multiple HER2-positive malignancies [47,48,49]. Trastuzumab deruxtecan, an HER2-directed ADC, has demonstrated substantial clinical activity in several HER2-expressing solid tumors and has also received tissue-agnostic regulatory approval for selected HER2-positive cancers [49,50,51,52]. However, HER2 amplification occurs in only a small subset of PDAC, and currently available pancreatic cancer-specific clinical data remain limited, with much of the evidence derived from basket studies or predominantly non-pancreatic tumor populations [49,50,51,52].

The integration of comprehensive molecular diagnostics has facilitated increasingly individualized therapeutic strategies based on genomic profiling and biomarker-guided treatment selection [14,15,16,25,53]. Nevertheless, important challenges remain, including the low prevalence of actionable alterations, molecular heterogeneity, acquired resistance, and limited therapeutic penetration within the characteristic pancreatic tumor microenvironment [11,12,13,23]. Continued advances in translational research, biomarker discovery, and rational combination strategies will therefore be essential to further improve outcomes in this highly lethal disease [19,54,55,56,57,58].

This narrative review focuses on three emerging therapeutic strategies that illustrate distinct approaches to precision oncology in PDAC: mutation-specific targeting with adagrasib, biomarker-driven immune checkpoint inhibition with dostarlimab, and HER2-directed ADC therapy with trastuzumab deruxtecan. Rather than providing a comprehensive review of all molecularly targeted therapies in pancreatic cancer, this article critically evaluates the biological rationale, mechanisms of action, clinical evidence, biomarker-guided patient selection, resistance mechanisms, and future perspectives for these three representative therapeutic platforms. Throughout this review, pancreatic cancer-specific clinical evidence is distinguished from tissue-agnostic or predominantly non-pancreatic evidence to facilitate appropriate interpretation of the currently available data.

A structured literature search was conducted in PubMed/MEDLINE, Scopus, and Web of Science to identify English-language publications published between January 2006 and May 2026. Search terms included combinations of “pancreatic cancer,” “pancreatic ductal adenocarcinoma,” “adagrasib,” “dostarlimab,” “trastuzumab deruxtecan,” “KRAS G12C,” “HER2,” “immune checkpoint inhibitors,” “antibody–drug conjugates,” “targeted therapy,” “precision medicine,” and “combination therapy.” Eligible publications included phase II and III clinical trials, randomized controlled trials, basket studies, registration-directed investigations, prospective and retrospective cohort studies, translational studies, systematic reviews, meta-analyses, and relevant international clinical practice guidelines. Priority was given to pivotal and regulatory-supporting studies evaluating KRAS-targeted therapy, immune checkpoint blockade in dMMR/MSI-H tumors, HER2-directed therapies, and biomarker-guided treatment strategies, whereas selected real-world studies were included when they provided clinically meaningful evidence regarding efficacy, safety, resistance mechanisms, or therapeutic sequencing. Publications were selected according to their scientific relevance, methodological quality, and clinical significance. Although a structured search strategy was employed, this review was conducted as a narrative review rather than a formal systematic review.

**Figure 1:**
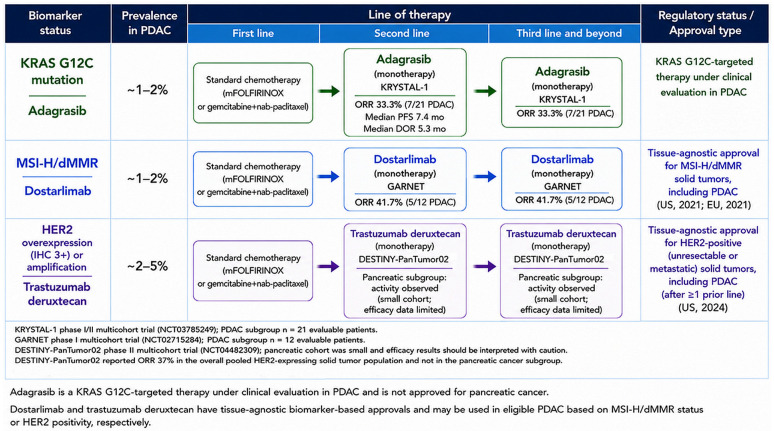
Schematic comparison overview of treatment selection in pancreatic cancer, integrating pivotal clinical trial data by biomarker status and line of therapy with the use of adagrasib, dostarlimab, and trastuzumab deruxtecan. The scheme reflects an original conceptual integration of the currently available evidence, designed in alignment with fundamental NCCN and ESMO recommendations, including biomarker-oriented treatment selection and therapeutic sequencing approaches. All abbreviations employed are defined in the Abbreviations section.

Figure 1 provides a schematic overview of precision therapeutic strategies in pancreatic cancer, integrating molecular profiling, biomarker-driven patient stratification, and selected targeted, immune, and ADC treatment approaches according to actionable molecular alterations, disease setting, and therapeutic sequencing.

## 2. Adagrasib

**Figure 2:**
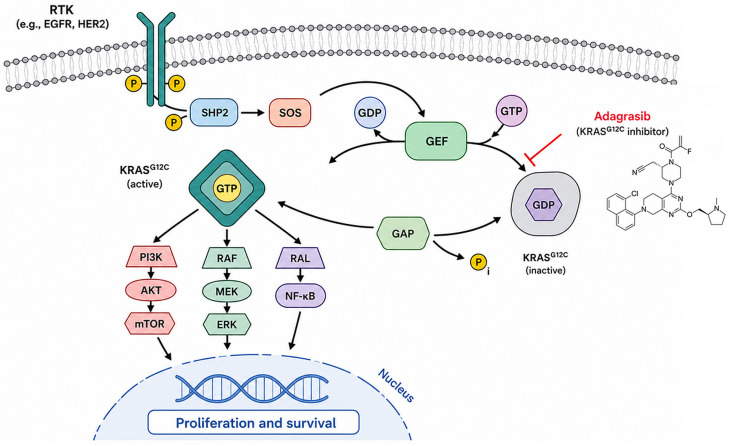
Schematic representation of the mechanism of action of adagrasib targeting KRAS G12C-mutated signaling pathways. Original schematic illustration created by the authors based on the mechanism described in [66]. Activation of receptor tyrosine kinases (RTKs), including EGFR and HER2, promotes downstream KRAS signaling through SHP2-, SOS-, and GEF-mediated nucleotide exchange, converting inactive GDP-bound KRAS G12C into its active GTP-bound form. Activated KRAS G12C subsequently stimulates multiple downstream proliferative and survival pathways, including the PI3K/AKT/mTOR, RAF/MEK/ERK, and RAL/NF-κB signaling cascades, ultimately promoting tumor cell proliferation and survival. GAP-mediated hydrolysis facilitates conversion of KRAS back to the inactive GDP-bound state. Adagrasib selectively binds and stabilizes inactive GDP-bound KRAS G12C, thereby preventing reactivation and suppressing oncogenic downstream signaling. All abbreviations employed are defined in the Abbreviations section.

Adagrasib is an orally bioavailable, irreversible covalent KRAS G12C inhibitor that selectively targets the inactive GDP-bound form of mutant KRAS, suppressing downstream MAPK and PI3K signaling involved in tumor proliferation and survival. The development of KRAS G12C inhibitors represents a major milestone in oncology, as KRAS was historically considered “undruggable” because of its high affinity for GTP/GDP and the absence of accessible binding pockets [31,34,59,60]. Although KRAS mutations occur in approximately 90% of PDAC, KRAS G12C is detected in only approximately 1–2% of cases, making adagrasib relevant to a highly selected molecular subgroup rather than the broader pancreatic cancer population [5,25,32,61]. Nevertheless, adagrasib provides important proof of principle that allele-specific KRAS inhibition can produce clinically meaningful activity in selected patients with heavily pretreated PDAC [23,33,62]. Recent reviews similarly emphasize that KRAS-directed therapy has reshaped the conceptual framework of pancreatic cancer precision oncology by transforming KRAS from an inaccessible oncogenic driver into a clinically actionable target in selected patients [37,63,64,65]. Figure 2 presents the mechanism of action of adagrasib.

Preclinical studies demonstrated that adagrasib, initially known as MRTX849, produces sustained inhibition of KRAS-dependent signaling through irreversible covalent binding to the cysteine residue generated by the G12C substitution [67,68]. In KRAS G12C-mutated in vitro and xenograft models, adagrasib suppressed ERK phosphorylation, inhibited tumor cell proliferation, and induced apoptosis [68,69]. Its pharmacokinetic profile, including prolonged systemic exposure, relatively long half-life, and extensive tissue distribution, was designed to support continuous target inhibition [69]. Additional translational studies suggested that KRAS blockade may modulate the tumor microenvironment, increase antigen presentation and T-cell infiltration, and potentially enhance sensitivity to immune checkpoint inhibition, although these findings remain primarily preclinical and require clinical validation in PDAC [70,71,72]. Medicinal chemistry studies further support the importance of covalent binding, allele selectivity, and optimized drug-like properties for durable suppression of mutant KRAS signaling [73,74].

The main clinical evidence for adagrasib derives from KRYSTAL-1 (NCT03785249), a phase I/II multicohort trial evaluating adagrasib monotherapy and combination regimens in advanced KRAS G12C-mutated solid tumors [75]. Early dose-escalation data showed manageable toxicity and antitumor activity across several malignancies, including non-small-cell lung cancer, colorectal cancer, and pancreatic cancer [75,76,77]. Importantly, the pancreatic cancer population represented a small subgroup within a broader basket-trial design; therefore, disease-specific conclusions should be interpreted cautiously [75,76,77,78]. Nevertheless, responses in this refractory PDAC subgroup were clinically notable given the limited efficacy of later-line chemotherapy and the historical absence of effective targeted options for KRAS-mutated pancreatic cancer [5,76,77,78]. Broader systematic analyses indicate that KRAS G12C inhibitors can produce meaningful responses across selected solid tumors, although efficacy varies by tumor lineage, co-alterations, and resistance biology [79,80].

Updated mature results from KRYSTAL-1 demonstrated an objective response rate (ORR) of 33.3% (7/21 evaluable patients) in the pancreatic cancer subgroup receiving adagrasib monotherapy, with a median progression-free survival of 7.4 months and a median duration of response of 5.3 months [33]. These data supersede earlier interim conference reports and currently provide the most informative pancreatic cancer-specific estimate of adagrasib activity. However, the findings remain limited by the small denominator, nonrandomized design, and selected study population. Accordingly, the results should be regarded as encouraging but not definitive evidence of benefit in KRAS G12C-mutated PDAC [33,75,77,78,81,82,83]. Although responses appeared relatively durable in some patients and support KRAS G12C as a biologically relevant dependency in selected pancreatic tumors, the rarity of this biomarker and the limited size of the available cohort underscore the need for cautious interpretation and prospective validation [62,75,82,84,85].

Several limitations remain important. KRYSTAL-1 was not designed as a pancreatic cancer-specific registration trial, and the PDAC cohort was substantially smaller than the lung or colorectal cancer cohorts [75,76,77]. This limits statistical precision, increases uncertainty around efficacy estimates, and restricts generalizability to routine clinical practice [76,77,78]. In addition, enrolled patients were highly selected and generally treated in specialized academic settings, while intratumoral heterogeneity and co-occurring genomic alterations may influence both response durability and resistance evolution [76,77,78,86,87,88]. These issues underscore the need for broader molecular screening, real-world validation, and prospective studies specifically focused on KRAS G12C-mutated pancreatic cancer [65,85].

Acquired resistance is a major barrier to durable benefit from adagrasib and related KRAS G12C inhibitors. Reported mechanisms include secondary KRAS alterations, bypass pathway activation, RTK-mediated signaling reactivation, epithelial–mesenchymal transition, and adaptive feedback through EGFR, SHP2, MEK, or PI3K signaling [87,88,89,90,91]. Because PDAC is characterized by extensive stromal complexity and signaling redundancy, monotherapy KRAS inhibition may be insufficient for long-term disease control in many patients [87,88,89,90,91,92,93]. Contemporary resistance-focused analyses further emphasize that adaptive pathway reactivation, lineage plasticity, and tumor-specific feedback signaling are central barriers to durable benefit from KRAS-targeted monotherapy [80,92,93]. These observations support rational combinations with EGFR inhibitors, SHP2 inhibitors, MEK inhibitors, immunotherapy, chemotherapy, or next-generation KRAS-directed agents [88,89,90,91,93].

Combination strategies involving adagrasib are therefore under active investigation. In KRYSTAL-1, adagrasib combined with cetuximab showed clinically meaningful activity in colorectal cancer, supporting the concept that dual KRAS and EGFR blockade can attenuate adaptive pathway reactivation [75,94,95]. However, pancreatic cancer-specific combination data remain limited, and most available clinical evidence for adagrasib combinations is extrapolated from non-pancreatic tumor types [75,94,95]. Preclinical data support further evaluation of EGFR, SHP2, SOS1, MEK, and PI3K pathway co-inhibition in PDAC, but these strategies require prospective validation in pancreatic cancer-specific cohorts [88,89,90,91,93,96].

Immunotherapeutic combinations are also biologically plausible. Preclinical evidence suggests that KRAS inhibition may increase antigen presentation, reduce immunosuppressive cytokine signaling, and enhance T-cell infiltration [70,71,72]. KRYSTAL-1 included exploratory cohorts combining adagrasib with pembrolizumab, but available efficacy data are derived mainly from lung cancer rather than pancreatic cancer populations [75]. Therefore, the relevance of this approach to PDAC remains uncertain, particularly given the typically immune-excluded and stromal-rich pancreatic tumor microenvironment [37,64,71,72]. Further biomarker-driven studies are required to determine whether KRAS inhibition can meaningfully sensitize pancreatic tumors to checkpoint blockade.

Additional future strategies include combinations with chemotherapy and next-generation KRAS pathway inhibitors. Preclinical pancreatic cancer models suggest that KRAS inhibition may enhance sensitivity to cytotoxic therapy through modulation of tumor metabolism and proliferative signaling [88,91]. Because KRAS G12C occurs in only a small fraction of PDAC, broader approaches targeting KRAS G12D, pan-RAS signaling, KRAS degradation, or downstream pathway dependencies may ultimately have greater applicability in pancreatic cancer [90,97,98,99,100,101,102,103,104]. Recent preclinical work with direct KRAS inhibitors further illustrates the complexity of allele-specific signaling suppression and context-dependent antiproliferative responses across tumor models [103,104]. In this context, adagrasib should be viewed not only as a therapeutic option for the rare KRAS G12C-mutated subgroup, but also as a translational model for broader KRAS-directed precision strategies in PDAC [64,65,98,100].

From a safety perspective, adagrasib has generally shown a manageable toxicity profile consistent with other KRAS G12C inhibitors [75,76,77]. Common adverse events include nausea, diarrhea, vomiting, fatigue, decreased appetite, hepatotoxicity, and QT interval prolongation [75,76,77,105,106]. Most events are grade 1–2 and manageable with supportive care, treatment interruption, or dose reduction [75,76,105,106]. Gastrointestinal toxicity is particularly relevant in PDAC, where patients frequently have baseline nutritional compromise, weight loss, and cancer-related cachexia [5,75]. Hepatic enzyme elevations and cumulative toxicity may become especially important when adagrasib is used in combination regimens [75,76,79]. Systematic analyses of KRAS G12C inhibitors similarly indicate that gastrointestinal, hepatic, and treatment-interruption events require careful monitoring, particularly when these agents are moved into combination strategies or frailer gastrointestinal cancer populations [79].

Clinically, adagrasib illustrates the growing importance of comprehensive molecular profiling in advanced PDAC. Although KRAS G12C mutations are rare, their identification can meaningfully alter treatment options for selected patients [25,32,61]. This supports broad next-generation sequencing rather than limited hotspot testing alone, particularly in advanced disease where standard therapeutic options remain limited [5,25,61,65,85]. At the same time, the rarity of KRAS G12C creates practical challenges for trial enrollment and implementation, emphasizing the need for multicenter screening initiatives, basket-trial designs, and disease-specific validation [25,32,65,85].

The broader clinical significance of adagrasib may therefore extend beyond its direct activity in KRAS G12C-mutated PDAC. Its development has revitalized efforts to target other KRAS alleles, particularly KRAS G12D, which represents the dominant KRAS subtype in pancreatic cancer [32,90,97]. Furthermore, adagrasib has demonstrated that direct KRAS inhibition can produce objective responses in gastrointestinal malignancies previously considered resistant to targeted therapy [62,77,82]. Nevertheless, resistance mechanisms, molecular heterogeneity, and limited patient eligibility remain major barriers to long-term disease control, highlighting the importance of combination strategies and adaptive therapeutic sequencing [87,88,89,90,91]. The ongoing development of KRAS G12D inhibitors, pan-RAS inhibitors, covalent KRAS-directed compounds, KRAS degraders, and rational combination approaches may therefore extend the conceptual impact of adagrasib to a much broader pancreatic cancer population in future therapeutic algorithms [74,96,101,102].

Taken together, adagrasib represents a clinically significant advance in precision oncology for pancreatic cancer by demonstrating that direct KRAS inhibition can produce meaningful antitumor activity in molecularly selected PDAC patients. Current evidence remains based on small, nonrandomized cohorts, and conclusions should therefore remain appropriately cautious [77,78,81,82,83]. Future progress will depend on prospective validation, an improved understanding of resistance mechanisms, optimization of rational combination regimens, and development of KRAS-targeted strategies applicable beyond the rare KRAS G12C subgroup, particularly KRAS G12D-driven disease. Accordingly, adagrasib should be viewed not only as an allele-specific intervention for KRAS G12C-mutated PDAC, but also as a translational bridge toward broader KRAS-directed precision strategies capable of addressing more prevalent pancreatic cancer genotypes [64,65,98,100].

Table 1 summarizes treatment-emergent adverse events (TEAEs) and their management strategies for adagrasib, while Table 2 outlines the major pivotal clinical trials and selected emerging studies of adagrasib in pancreatic cancer.

## 3. Dostarlimab

**Figure 3:**
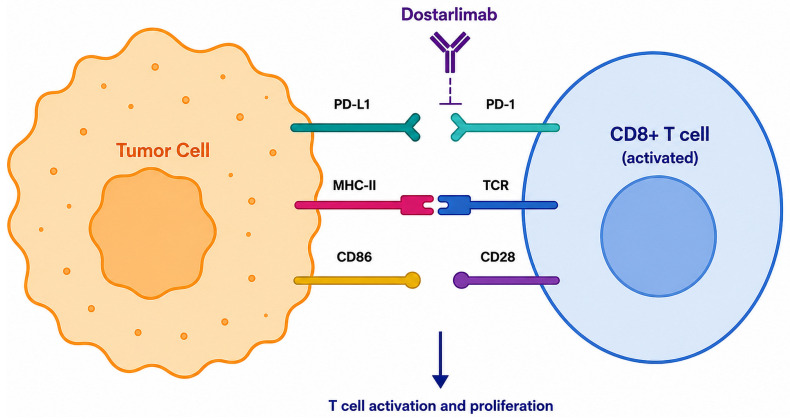
Schematic illustration of the mechanism of action of dostarlimab targeting the PD-1 immune checkpoint pathway. Original schematic illustration created by the authors based on the mechanism described in [123]. Tumor cells expressing PD-L1 interact with PD-1 receptors on CD8^+^ T cells, leading to suppression of antitumor immune responses and T-cell exhaustion. Dostarlimab, a monoclonal antibody directed against PD-1, blocks the PD-1/PD-L1 interaction, thereby restoring T-cell activation, proliferation, and cytotoxic antitumor activity. Concurrent antigen presentation through the major histocompatibility complex (MHC-II)/T-cell receptor (TCR) interaction and co-stimulatory signaling via CD86/CD28 further contribute to effective immune activation. In combination, blockade of the PD-1 signaling axis enhances immune-mediated recognition and elimination of tumor cells. All abbreviations employed are defined in the Abbreviations section.

Dostarlimab is a humanized IgG4 monoclonal antibody targeting programmed cell death protein 1 (PD-1), restoring antitumor immunity by blocking interactions between PD-1 and its ligands PD-L1 and PD-L2 [113,114,115,116]. By inhibiting this immune checkpoint pathway, dostarlimab enhances cytotoxic T-cell activation, proliferation, and tumor-directed immune responses, thereby counteracting tumor-induced immune evasion [40,114,115,116]. Similar to other PD-1 inhibitors, its biological activity is greatest in tumors characterized by dMMR, MSI-H, or high tumor mutational burden, which are associated with increased neoantigen generation and enhanced immunogenicity [5,40,114,115,116]. Although PDAC is generally considered an immunologically “cold” malignancy, approximately 1% of pancreatic cancers harbor dMMR/MSI-H alterations and may benefit from immune checkpoint inhibition [5,24,25,117,118]. Consequently, comprehensive molecular profiling remains essential for identifying this rare but clinically relevant subgroup [24,25,117,118]. Recent reviews further emphasize that dostarlimab belongs to the expanding class of PD-1/PD-L1-directed immunotherapies that have reshaped biomarker-driven oncology by enabling durable responses in selected immune-responsive tumor phenotypes [119,120,121,122]. Figure 3 illustrates the mechanism of action of dostarlimab.

The principal clinical evidence for dostarlimab derives from the phase I GARNET study (NCT02715284), an open-label, multicenter, nonrandomized basket trial evaluating dostarlimab monotherapy in patients with recurrent or advanced dMMR/MSI-H solid tumors [124,125,126,127]. The study adopted a tissue-agnostic design based on molecular phenotype rather than tumor histology, reflecting the evolving paradigm of biomarker-driven immunotherapy [124,125,126,127,128,129,130,131]. Early dose-escalation and expansion cohorts demonstrated durable antitumor activity and a manageable safety profile across multiple tumor types, particularly endometrial and gastrointestinal malignancies [124,125,126,127,128]. This tissue-agnostic rationale is particularly relevant in pancreatic cancer, where actionable molecular subsets are uncommon but increasingly therapeutically important [129,130,131].

Within GARNET, the pancreatic cancer subgroup consisted of only 12 patients. Updated analyses reported ORR of 41.7% (5/12 patients), with durable responses observed in some individuals during extended follow-up [125,126,132]. However, these findings should be interpreted cautiously because they originate from an extremely small pancreatic cancer subgroup within a larger basket trial. Consequently, the efficacy estimate is associated with wide confidence intervals, substantial sampling variability, limited statistical precision, and potential subgroup-related bias, and should therefore be considered exploratory rather than definitive evidence of efficacy in PDAC [125,126,132]. Although responses across multiple tumor types support the biological relevance of dMMR as a predictive biomarker independent of tissue origin, extrapolation to pancreatic cancer should remain cautious because PDAC has distinct stromal biology and a profoundly immunosuppressive tumor microenvironment [125,126,127,128,129,132].

Importantly, the regulatory approval of dostarlimab was based on tissue-agnostic efficacy across dMMR solid tumors rather than pancreatic cancer-specific clinical trials [125,126,127,128,129,132]. Clinical experience from endometrial and colorectal cancer supports the broader principle that dostarlimab can induce meaningful responses in dMMR/MSI-H tumors when an immune-sensitive phenotype is present [133,134,135]. Nevertheless, PDAC-specific clinical evidence remains limited. Therefore, the current role of dostarlimab in pancreatic cancer is supported primarily by biomarker-driven tissue-agnostic evidence, while dedicated pancreatic cancer studies remain necessary to define its efficacy more precisely [125,126,127,128,129,132,133,134,135].

Evidence from pembrolizumab and other PD-1 inhibitors has similarly established proof of concept that dMMR/MSI-H pancreatic cancers can achieve durable responses despite the generally poor immunogenicity of unselected PDAC [41,136,137,138]. These observations support the concept that immune checkpoint inhibition represents an effective therapeutic strategy in this rare molecular subset, although prospective pancreatic cancer-specific datasets remain limited [41,124,125,126,127]. The rarity of MSI-H/dMMR pancreatic cancer also complicates trial enrollment and statistical interpretation, leading many studies to rely on basket-trial designs rather than dedicated PDAC cohorts [124,125,126,127,129,131].

The immunologically suppressive biology of PDAC remains a major obstacle to broader application of checkpoint inhibitors. PDAC is characterized by dense desmoplastic stroma, low baseline T-cell infiltration, abundant immunosuppressive myeloid populations, and complex cytokine-mediated immune suppression, all of which contribute to resistance to immunotherapy [13,139,140,141]. Consequently, therapeutic benefit appears largely restricted to biomarker-selected tumors with dMMR/MSI-H or other hypermutated phenotypes [117,118,139,140,141,142,143,144,145]. Even within biomarker-selected populations, responses may remain heterogeneous, suggesting that additional genomic, epigenetic, and microenvironmental factors influence immunotherapy sensitivity [140,141,142]. Structural and mechanistic studies of the PD-1/PD-L1/PD-L2 axis further support effective ligand blockade at the receptor interface as the central pharmacologic basis of dostarlimab-mediated immune activation [143,144,145].

To overcome intrinsic immune resistance, multiple combination strategies are being investigated, including chemotherapy, radiotherapy, dual checkpoint blockade, cancer vaccines, stromal modulation, CD40 agonists, and KRAS-targeted therapies [140,141,142,143,144,145,146,147,148,149,150]. However, it should be emphasized that most available clinical data involve pembrolizumab, nivolumab, or durvalumab rather than dostarlimab itself [140,141,142,143,144,145,146,147]. Accordingly, the rationale for combination therapy with dostarlimab in PDAC remains largely extrapolated from mechanistic studies and clinical experience with other immune checkpoint inhibitors rather than from pancreatic cancer-specific clinical trials. Broader immunotherapy literature suggests that PD-1/PD-L1 blockade may be most effective when integrated with rational combination partners capable of enhancing antigen presentation, T-cell priming, immune infiltration, and reversal of suppressive tumor microenvironmental signaling [149,150].

Chemotherapy-based combinations are particularly attractive because cytotoxic agents may increase neoantigen release, promote immunogenic cell death, and reduce immunosuppressive stromal barriers [39,146,147,148]. However, clinical outcomes with chemo-immunotherapy in unselected pancreatic cancer populations have generally been disappointing, with only modest improvements observed in most studies [39,146]. These findings suggest that biomarker-driven patient selection remains essential for effective integration of checkpoint inhibitors into pancreatic cancer treatment paradigms. In this context, dostarlimab may ultimately prove most clinically valuable within carefully selected molecular subsets rather than as a broadly applicable immunotherapeutic strategy for all PDAC patients [117,118,119,121,151].

Additional biomarkers, including tumor mutational burden, POLE/POLD1 alterations, immune gene-expression signatures, tertiary lymphoid structures, and circulating immune biomarkers, are currently under investigation but remain insufficiently validated in pancreatic cancer [125,126,130,131,140,141,142]. Notably, the GARNET study also included patients with POLE-altered tumors, reflecting increasing recognition that hypermutated phenotypes may predict enhanced sensitivity to PD-1 blockade irrespective of traditional MSI classification [125,126]. The expanding experience with tissue-agnostic therapies suggests that biomarker-defined immunotherapy approaches may become increasingly refined as molecular profiling expands beyond MSI/MMR testing alone [130,131].

From a safety perspective, dostarlimab demonstrates a toxicity profile consistent with other PD-1 inhibitors [125,126,127,128,132]. The most common adverse events include fatigue, diarrhea, nausea, rash, pruritus, arthralgia, and infusion-related reactions, whereas immune-mediated toxicities may involve endocrinopathies, hepatitis, colitis, pneumonitis, nephritis, and dermatologic reactions [125,126,127,128,132]. Most immune-related adverse events are manageable with early recognition, corticosteroid therapy, and treatment interruption when appropriate [125,126,127,128,132]. Severe immune-related toxicities occur less frequently but remain clinically important, particularly in frail pancreatic cancer patients with poor baseline nutritional and performance status [5,39]. Integrated safety and immunogenicity analyses have also demonstrated a favorable immunogenicity profile, supporting continued clinical development across multiple tumor types [152,153,154].

An additional clinically important consideration involves the durability of immunotherapy responses. Although only a minority of pancreatic cancer patients appear eligible for dostarlimab monotherapy, responders may experience prolonged disease control substantially exceeding expectations associated with standard chemotherapy [41,136,137]. This observation highlights one of the defining advantages of immune checkpoint inhibition: the potential for durable immune-mediated tumor suppression rather than transient cytotoxic responses alone [40,44,114,115,116]. Durable responses observed in other dMMR/MSI-H solid tumors, including endometrial and rectal cancer, nevertheless reinforce the biological plausibility of sustained benefit in appropriately selected pancreatic cancer patients [133,134,135].

The regulatory development of dostarlimab also reflects the broader evolution of tissue-agnostic oncology. The approval of PD-1 inhibitors for dMMR solid tumors marked a major conceptual shift away from organ-specific treatment paradigms toward biomarker-driven therapeutic selection [41,132]. In pancreatic cancer, where conventional therapeutic progress has historically been limited, this approach has particular significance because even rare actionable molecular subsets may provide opportunities for highly effective personalized therapy [5,24,25,117,118]. Consequently, universal molecular testing—including assessment of MSI/MMR status, BRCA alterations, KRAS variants, HER2 expression, NTRK fusions, and additional genomic biomarkers—is increasingly recommended in advanced pancreatic cancer management [24,25,40,117,118]. Current discussions of tumor-agnostic precision medicine further emphasize that molecularly selected treatment indications can be especially important in pancreatic cancer, where conventional options remain limited and rare biomarkers may meaningfully change therapeutic strategy [129,130,155].

Nevertheless, several unresolved challenges remain. The low incidence of dMMR/MSI-H pancreatic cancer limits the feasibility of large dedicated randomized trials, and most current evidence derives from basket studies with heterogeneous tumor populations [41,124,125,126,127]. Furthermore, resistance to immune checkpoint inhibition may emerge through multiple mechanisms, including loss of antigen presentation, interferon signaling alterations, T-cell exhaustion, and adaptive immune suppression within the tumor microenvironment [139,140,141,142,156,157]. These resistance pathways likely contribute to the limited efficacy of checkpoint inhibition in the majority of pancreatic cancers and reinforce the need for rational combination strategies capable of modulating stromal and immune barriers. Broader checkpoint blockade literature similarly highlights that resistance to PD-1/PD-L1 inhibition is multifactorial and often requires combinatorial strategies rather than simple escalation of single-agent immunotherapy [149,158].

Dostarlimab represents an important example of tissue-agnostic precision immuno-oncology in pancreatic cancer. Although only a small proportion of PDAC patients are eligible because of the rarity of dMMR/MSI-H disease, the available evidence indicates that carefully selected patients may achieve clinically meaningful and occasionally durable responses to PD-1 blockade [24,25,117,118,125,126,127,128]. Nevertheless, current pancreatic cancer-specific evidence remains limited to small nonrandomized cohorts and should therefore be interpreted cautiously. Future progress will depend on broader molecular testing, refinement of predictive biomarkers beyond MSI/MMR status, prospective pancreatic cancer-specific studies, and development of rational combination strategies capable of overcoming the immunosuppressive pancreatic tumor microenvironment [129,130,131,152,153,154,155,156,157,158,159,160]. Careful monitoring and guideline-based management of immune-related adverse events remain essential for safe clinical implementation, particularly as combination immunotherapeutic strategies become increasingly complex [159,160]. Dostarlimab should be viewed not as a broadly applicable therapy for unselected PDAC, but as a clinically meaningful precision immunotherapy option within rare immune-responsive pancreatic cancer subsets and as a model for future tissue-agnostic biomarker-driven therapeutic development [119,120,129,131].

Table 3 summarizes TEAEs and their management strategies for dostarlimab, while Table 4 outlines the major pivotal clinical trials and selected emerging studies of dostarlimab in pancreatic cancer.

## 4. Trastuzumab Deruxtecan

**Figure 4:**
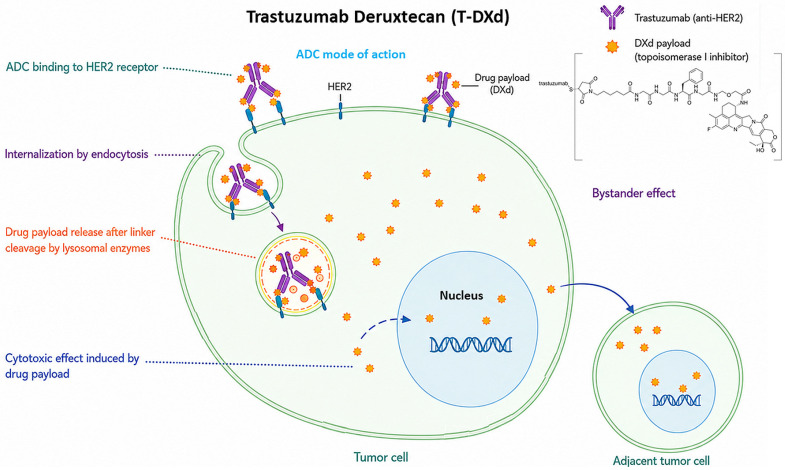
Schematic representation of the mechanism of action of trastuzumab deruxtecan (T-DXd), an HER2-directed ADC. Original schematic illustration created by the authors based on the mechanism described in [180]. T-DXd consists of a monoclonal anti-HER2 antibody (trastuzumab) linked to a potent topoisomerase I inhibitor payload (DXd) through a cleavable linker. Following selective binding of the ADC to HER2 receptors expressed on the tumor cell surface, the complex undergoes receptor-mediated internalization via endocytosis. Subsequent lysosomal degradation and linker cleavage release the cytotoxic DXd payload into the intracellular compartment. The released payload induces DNA damage and cytotoxicity, ultimately promoting tumor cell death. Due to membrane permeability of the released payload, neighboring tumor cells with variable or lower HER2 expression may also be affected through a bystander effect, thereby enhancing antitumor activity within heterogeneous tumor microenvironments. All abbreviations employed are defined in the Abbreviations section.

Trastuzumab deruxtecan (T-DXd; DS-8201a) is an HER2-directed ADC consisting of a humanized anti-HER2 monoclonal antibody derived from trastuzumab, a cleavable tetrapeptide linker, and a potent topoisomerase I inhibitor payload derived from exatecan [49,51,169,170]. Compared with earlier HER2-directed ADCs, T-DXd was engineered with a higher drug-to-antibody ratio and a membrane-permeable payload capable of producing a clinically relevant bystander effect, enabling the elimination of adjacent tumor cells with heterogeneous HER2 expression [51,170,171,172]. These properties are particularly relevant in pancreatic cancer, where HER2 amplification or overexpression occurs in approximately 1–7% of PDAC and frequently demonstrates substantial intratumoral heterogeneity [5,14,25,50,173]. Recent reviews further highlight T-DXd as a major advance in HER2-directed precision oncology because its molecular design may overcome limitations associated with heterogeneous antigen expression and inefficient intracellular payload delivery [174,175,176]. Mechanistically, T-DXd binds HER2-expressing tumor cells, undergoes receptor-mediated internalization, and releases its membrane-permeable cytotoxic payload following lysosomal linker cleavage [51,169,170,171,172]. The released topoisomerase I inhibitor induces DNA damage and apoptotic cell death while also producing bystander cytotoxicity in neighboring HER2-low cells [171,172]. In addition, the trastuzumab backbone retains canonical HER2-targeting functions, including inhibition of HER2 signaling and antibody-dependent cellular cytotoxicity [49,169,170]. Experimental studies further suggest that extracellular payload release, improved ADC internalization, and immunomodulatory effects may contribute to the therapeutic activity of T-DXd and related HER2-directed ADCs [177,178,179]. Figure 4 illustrates the mechanism of action of trastuzumab deruxtecan.

Clinical development of T-DXd initially focused on HER2-positive breast and gastric cancers, where early studies demonstrated substantial and durable antitumor activity, ultimately establishing the agent as an important HER2-directed therapy across multiple solid tumors [181,182,183,184,185,186,187,188,189,190]. The phase I first-in-human trial reported durable responses across HER2-expressing malignancies, while DESTINY-Breast01 established T-DXd as a major therapeutic advance in metastatic HER2-positive breast cancer [181,182]. Subsequent clinical experience in breast, gastric, and lung cancer further confirmed the broad antitumor potential of T-DXd across HER2-expressing or HER2-mutated malignancies, providing the biological and clinical rationale for evaluating T-DXd in less common HER2-positive malignancies, including pancreatic cancer [184,185,186,187,188,189,190].

In pancreatic cancer, the rationale for HER2-directed therapy derives from the recognition that a subset of PDACs demonstrate HER2 amplification, overexpression, or activating HER2 mutations associated with oncogenic signaling dependence [5,14,25,50,173]. Although HER2 alterations occur less frequently in PDAC than in breast or gastric cancer, emerging genomic and real-world tumor-agnostic studies suggest that HER2-positive pancreatic tumors may represent a clinically actionable subgroup potentially sensitive to HER2-targeted agents [50,173,191,192,193,194,195]. Earlier trastuzumab-based combinations in pancreatic cancer produced inconsistent results, partly because of limited biomarker standardization, small sample sizes, and inadequate molecular selection strategies [191,192,193]. Consequently, the development of T-DXd has renewed interest in HER2-directed precision therapeutics for pancreatic cancer.

The principal pancreatic cancer evidence derives from the phase II DESTINY-PanTumor02 study (NCT04482309), an open-label, multicenter basket trial evaluating T-DXd in HER2-expressing solid tumors [196,197,198,199]. The study demonstrated clinically meaningful tumor-agnostic activity across several HER2-positive malignancies, including pancreatic cancer, although PDAC represented only a relatively small subgroup because of the rarity of HER2-positive disease [196,197,198,199]. Related HER2-directed studies in biliary tract and other gastrointestinal cancers provide additional support for extending T-DXd evaluation across HER2-positive digestive system malignancies, although tumor-specific efficacy remains variable [200,201].

Updated analyses demonstrated antitumor activity in HER2-expressing pancreatic cancer, with responses observed predominantly in tumors demonstrating strong HER2 overexpression (IHC 3+) [197,198,199,202,203]. However, pancreatic cancer-specific efficacy remained more modest than that observed in breast or gastric cancer, and interpretation should remain cautious because the pancreatic cohort was small, nonrandomized, heavily pretreated, and heterogeneous with respect to prior therapies and biomarker assessment [197,198,199,202,203]. Clinicopathologic analyses from breast cancer also suggest that baseline tumor biology, HER2 expression intensity, and disease characteristics may influence T-DXd efficacy, supporting the need for refined biomarker interpretation in pancreatic cancer [204].

Importantly, the currently available pancreatic cancer evidence should be distinguished from the broader tumor-agnostic experience with T-DXd. Although clinical activity across multiple HER2-positive malignancies supported tissue-agnostic regulatory approval and reinforced HER2 as a therapeutically actionable biomarker, pancreatic cancer-specific data remain limited and originate primarily from basket studies rather than dedicated PDAC clinical trials [196,197,198,199,202,203]. Consequently, current evidence should be considered encouraging but exploratory, and prospective pancreatic cancer-specific studies remain necessary to better define the efficacy of T-DXd in PDAC. These limitations also highlight the need for pancreatic cancer-specific HER2 testing harmonization and prospective validation of HER2 thresholds most predictive of benefit from ADC therapy [174,194].

Additional support for HER2-directed therapy comes from DESTINY-PanTumor01 and other pan-tumor studies evaluating HER2-mutated or HER2-amplified solid tumors [205]. Although pancreatic cancer representation remained limited, these studies further support the concept that HER2 alterations—including amplification, overexpression, and activating mutations—may each define therapeutically actionable but biologically distinct molecular subsets [175,189,194,205,206]. Real-world molecular profiling studies likewise suggest that HER2-positive pancreatic cancers may be underrecognized without comprehensive genomic testing [14,25,50,206].

Preclinical studies also support the biological rationale for HER2-directed ADC therapy in pancreatic cancer. Experimental models demonstrated potent activity against HER2-overexpressing pancreatic tumor cells while maintaining efficacy in heterogeneous HER2-expression settings through the bystander effect [51,171,172]. This property may be particularly relevant in PDAC, where intratumoral heterogeneity can limit the effectiveness of conventional HER2-targeted therapies [5,25,50,173]. Nevertheless, resistance mechanisms remain incompletely understood and may involve HER2 downregulation, impaired ADC internalization, drug efflux activation, alterations in DNA damage response pathways, or tumor microenvironment-mediated resistance [177,207,208,209,210]. Recent experimental work also suggests that EGFR-directed antibodies may promote HER2 ADC internalization and efficacy, and that combined KRAS–MAPK pathway inhibition with HER2-directed ADC therapy may enhance antitumor activity in pancreatic cancer models [177,210].

Combination strategies involving T-DXd are currently under investigation and include chemotherapy, ICIs, tyrosine kinase inhibitors, additional HER2-directed therapies, KRAS-directed therapy, and stromal-modulating approaches [207,208,209,210,211]. Preclinical evidence suggests that HER2-directed ADCs may enhance immunogenic cell death and therefore provide a rationale for combinations with PD-1/PD-L1 blockade [208,209,211]. However, pancreatic cancer-specific combination data remain scarce, and most current concepts are extrapolated from breast, gastric, or lung cancer studies rather than prospective PDAC trials [207,208,209,210,211]. Dual-function ADC platforms incorporating cytotoxic payloads and immune-activating components further illustrate how next-generation HER2-directed conjugates may evolve toward simultaneous tumor killing and immune modulation [176,178,179].

From a safety perspective, trastuzumab deruxtecan demonstrates a toxicity profile characteristic of topoisomerase I inhibitor-containing ADCs [196,197,198,199,202,203]. Common adverse events include nausea, fatigue, vomiting, myelosuppression, anemia, decreased appetite, and alopecia [181,182,183,184,185,186]. The most clinically important toxicity is interstitial lung disease (ILD)/pneumonitis, which requires prompt recognition, treatment interruption, and corticosteroid therapy because fatal cases have been reported [182,184,185,186,212]. Although ILD incidence in DESTINY-PanTumor02 appeared generally consistent with previous studies, interpretation remains limited by the relatively small pancreatic cancer cohort [197,198,199]. Gastrointestinal toxicities are also clinically relevant in pancreatic cancer patients, who frequently have baseline cachexia, nutritional compromise, and treatment-related gastrointestinal dysfunction [172,212]. Reviews focusing on gastrointestinal oncology similarly emphasize careful monitoring for ILD, hepatic toxicity, myelosuppression, and gastrointestinal adverse events when T-DXd is administered to medically fragile patients [213,214,215].

Appropriate patient selection remains critical because the greatest benefit has been observed in tumors with high HER2 expression, particularly IHC 3+ disease [197,198,199,202,203]. However, HER2 testing in pancreatic cancer is considerably less standardized than in breast or gastric cancer owing to intratumoral heterogeneity, incomplete or basolateral membranous staining patterns, variable HER2 protein expression, and differences in scoring methodology [25,50,173,216]. Consequently, HER2 assessment should ideally incorporate experienced gastrointestinal pathology review together with confirmatory in situ hybridization or comprehensive genomic profiling in selected equivocal cases [25,50,173,216]. Standardization of HER2 testing specifically for PDAC remains an important unmet need. Pharmacokinetic and pharmacogenomic studies may also contribute to individualized dosing, toxicity prediction, and optimization of ADC exposure in heterogeneous solid tumor populations [217,218,219].

The broader significance of T-DXd extends beyond pancreatic cancer itself. The success of T-DXd has accelerated development of next-generation ADCs targeting HER2 and other molecular alterations across solid tumors, highlighting the expanding role of ADCs in precision oncology and personalized treatment strategies [47,51,171,172,220]. More broadly, T-DXd exemplifies the evolving paradigm of biomarker-driven tissue-agnostic oncology, in which therapeutically actionable molecular alterations may supersede conventional organ-specific classifications [196,197,198,199,202,203,205]. This concept is particularly important in pancreatic cancer, where historically limited therapeutic advances have increasingly given way to molecularly stratified treatment approaches involving BRCA mutations, KRAS variants, MSI-H/dMMR status, NTRK fusions, and HER2 alterations [5,25,50,173]. The first tumor-agnostic approval of an HER2-directed ADC has further strengthened interest in expanding HER2-based therapeutic selection across rare solid tumor subsets identified through molecular profiling [175,195,221].

Despite these advances, several unresolved questions remain. The rarity of HER2-positive pancreatic cancer limits the feasibility of large randomized studies and contributes to reliance on basket-trial designs with heterogeneous patient populations [196,197,198,199,202,203]. Furthermore, optimal sequencing of T-DXd relative to chemotherapy, immunotherapy, and other targeted therapies remains undefined. Resistance mechanisms are also incompletely characterized, and the durability of benefit in pancreatic cancer appears less pronounced than in HER2-positive breast cancer [207,208,209,211]. Additionally, concerns regarding ILD and cumulative toxicity may become increasingly important as T-DXd is incorporated into multidrug regimens or used earlier in the disease course [182,184,185,186,212]. Future development will therefore require integrated biomarker refinement, prospective pancreatic cancer-specific cohorts, and translational studies examining ADC internalization, payload sensitivity, DNA damage response, immune activation, and resistance evolution [174,178,222].

Viewed together, trastuzumab deruxtecan represents a promising HER2-directed precision therapy for a small molecularly defined subgroup of pancreatic cancer patients. Current pancreatic cancer-specific evidence remains limited to relatively small, nonrandomized basket-trial cohorts and should therefore be interpreted cautiously [196,197,198,199,202,203]. Nevertheless, the observed activity supports HER2 as a therapeutically actionable biomarker in selected PDAC patients. Future progress will depend on standardized HER2 testing, refinement of predictive biomarkers beyond HER2 expression alone, prospective pancreatic cancer-specific clinical studies, optimization of rational combination strategies, and an improved understanding of resistance biology [174,176,207,208,209,210,211,212,213,214,215,216,217,218,219,220,221,222]. Together, these findings suggest that T-DXd should be viewed not only as a therapeutic option for carefully selected HER2-positive PDAC patients, but also as a model for the expanding role of ADCs in biomarker-driven precision oncology [174,176,214].

Table 5 summarizes TEAEs and their management strategies for trastuzumab deruxtecan, while Table 6 outlines the major pivotal clinical trials and selected emerging studies of trastuzumab deruxtecan in pancreatic cancer.

## 5. Future Perspectives on Precision Oncology and Clinical Management of Pancreatic Cancer

Future perspectives in pancreatic cancer increasingly center on the transition from empiric treatment algorithms toward biologically stratified precision oncology, integrating molecular profiling, biomarker-guided systemic therapy, adaptive sequencing, and multidisciplinary clinical management [5,14,231,232]. Despite advances in surgery, chemotherapy, radiotherapy, and supportive care, PDAC remains associated with poor long-term survival because of late diagnosis, early metastatic dissemination, aggressive tumor biology, stromal complexity, and therapeutic resistance [231,232]. Nevertheless, the identification of actionable molecular alterations—including KRAS G12C mutations, HER2 amplification, BRCA1/2- or PALB2-associated homologous recombination deficiency, MSI-H/dMMR status, NTRK fusions, NRG1 rearrangements, CLDN18.2 expression, and additional KRAS variants such as KRAS G12D and KRAS G12V—has created opportunities for increasingly individualized therapeutic approaches [24,25,26,231,232,233,234,235,236,237,238,239,240,241,242,243]. Emerging translational studies additionally suggest that modulation of autophagy, hypoxic adaptation, lipid metabolism, epithelial–mesenchymal transition, and cancer stemness pathways may further expand precision therapeutic strategies, while bioengineered nanoparticles, peptide-guided therapeutics, glycosylated chitosan platforms, and advanced antibody delivery systems illustrate the growing diversity of targeted drug-delivery technologies under investigation [234,235,236,237,238,239,240,241,242,243].

Although this review focuses on adagrasib, dostarlimab, and trastuzumab deruxtecan as representative examples of targeted KRAS inhibition, immune checkpoint blockade, and HER2-directed ADC therapy, future precision oncology in PDAC will extend well beyond these three agents. Broader development of PARP inhibitor strategies, KRAS G12D inhibitors, pan-RAS inhibitors, KRAS degraders, fusion-directed therapies, CLDN18.2-targeted agents, ADCs, and rational combination regimens will likely further reshape biomarker-driven treatment algorithms [197,231,232,233,234,235,236,237,238,239,240,241].

Surgical resection remains the cornerstone of curative-intent therapy for localized pancreatic cancer, particularly in resectable and selected borderline resectable disease [5,231,244]. Future surgical management will likely become increasingly integrated with response-adapted neoadjuvant strategies, molecular residual disease assessment, circulating tumor DNA (ctDNA), CA19-9 dynamics, radiographic response, and molecular signatures to improve patient selection and perioperative risk stratification [245,246,247,248,249,250]. The growing use of neoadjuvant systemic therapy reflects increasing recognition that biologically aggressive tumors frequently harbor occult micrometastatic disease before surgery, while patient-derived organoids and genetically engineered mouse models may further support individualized drug-sensitivity testing and biomarker validation in perioperative settings [245,246,247,248,249,250,251,252].

Adjuvant therapy also continues to evolve. Modified FOLFIRINOX remains one of the most effective postoperative regimens for fit patients after R0/R1 resection, while gemcitabine-capecitabine remains relevant in selected patients [253,254]. Future adjuvant strategies will likely incorporate ctDNA-guided risk assessment, molecular residual disease monitoring, and individualized escalation or de-escalation according to recurrence risk, treatment tolerance, and patient fitness [249,250,253,254,255].

In metastatic disease, systemic chemotherapy remains foundational for most patients lacking actionable molecular alterations [5,231,232]. FOLFIRINOX, gemcitabine plus nab-paclitaxel, and NALIRIFOX represent important multidrug options for fit patients, but toxicity continues to limit applicability in frail or elderly populations [8,9,10,256]. Future systemic treatment selection will require more precise integration of molecular biomarkers, pharmacogenomic predictors, geriatric assessment, nutritional status, performance status, patient preferences, and artificial intelligence-assisted clinical decision support. Nanotechnology-based drug delivery systems, thermosensitive hydrogels, and multifunctional nanoparticle formulations may further improve therapeutic delivery while reducing systemic toxicity [10,240,257,258,259].

Among precision strategies, homologous recombination deficiency remains one of the most clinically validated targets in PDAC. The POLO trial established maintenance olaparib as a biomarker-selected strategy in germline BRCA-mutated metastatic pancreatic cancer after platinum-based chemotherapy, although OS benefit remained less definitive [260,261,262]. Future work should clarify the role of PARP inhibitors beyond germline BRCA1/2 mutations, including PALB2 alterations, broader homologous recombination deficiency phenotypes, and combinations with immunotherapy, ATR inhibitors, or other DNA-damage response-targeted agents. CRISPR/Cas9-based therapeutic engineering, synthetic lethality approaches, and advanced gene-editing technologies may further expand biomarker-directed therapeutic opportunities [261,262,263,264,265,266].

KRAS-directed therapy represents another major future direction. Although KRAS G12C mutations occur in only a small minority of PDAC, adagrasib and sotorasib have demonstrated that direct KRAS inhibition is feasible in pancreatic cancer [23,32,33,81,267]. Because KRAS G12D is substantially more common in PDAC, next-generation KRAS G12D inhibitors, including MRTX1133 (Mirati Therapeutics), zoldonrasib (RMC-9805; Revolution Medicines), INCB161734 (Incyte), and setidegrasib (ASP3082; Astellas Pharma), may ultimately have broader clinical relevance [268,269,270,271,272,273]. However, early activity should be interpreted cautiously until confirmed in larger prospective trials. Combination strategies targeting adaptive resistance through SHP2, EGFR, MEK, PI3K, FAK, autophagy, or metabolic pathways may be required to achieve durable benefit [69,81,267,274,275].

HER2-directed therapy and ADC development also remain promising for molecularly selected PDAC. Although HER2 amplification or overexpression occurs in only a minority of cases, basket studies involving trastuzumab-based strategies and trastuzumab deruxtecan support HER2 as an actionable biomarker in selected patients [47,173,197]. Future progress will depend on harmonized HER2 testing, improved definition of predictive thresholds, a better understanding of HER2 heterogeneity, and prospective pancreatic cancer-specific validation. Advanced antibody engineering, biomimetic exosome delivery systems, and multifunctional targeted nanoplatforms may further improve therapeutic specificity and tumor penetration [47,173,197,241,276].

Immunotherapy will likely remain restricted to biomarker-selected pancreatic cancer subsets unless effective microenvironment-modulating combinations are developed. Rare MSI-H/dMMR pancreatic cancers may benefit from pembrolizumab or dostarlimab, supporting tissue-agnostic immunotherapy in selected patients [40,41,125]. However, unselected PDAC remains poorly responsive to checkpoint inhibition because of stromal desmoplasia, immune exclusion, low T-cell infiltration, and immunosuppressive signaling [12,13,147]. Future strategies may include combinations with chemotherapy, radiotherapy, vaccines, CD40 agonists, KRAS inhibitors, adoptive cellular therapies, oncolytic viruses, stromal-targeting approaches, and CRISPR-mediated immune modulation [125,147,156,277,278,279,280,281,282]. Biomarkers such as tumor mutational burden, tertiary lymphoid structures, immune gene-expression signatures, and ctDNA-based immune monitoring may help refine patient selection [41,125,156].

Rare fusion-driven pancreatic cancers further illustrate the value of comprehensive molecular profiling. NTRK fusion-positive PDAC may respond to larotrectinib or entrectinib, while NRG1 fusion-positive tumors have demonstrated sensitivity to zenocutuzumab and related HER3-targeted approaches [233,283,284,285]. Although these subsets are uncommon, their identification reinforces the need for broad next-generation sequencing rather than limited hotspot testing alone. Integration of microbiome characterization, metabolic phenotyping, and multi-omic profiling may further refine biologic subclassification and therapeutic personalization [233,283,284,285,286,287].

Radiotherapy, SBRT, and local ablative approaches may continue to evolve within multidisciplinary management, particularly for selected patients with locally advanced or borderline resectable disease [288,289,290,291,292]. Future integration with immunotherapy, DNA-damage response inhibitors, adaptive image guidance, molecular imaging, treatment-response biomarkers, and local ablative technologies such as irreversible electroporation may improve local control and personalize multimodal therapy [288,289,290,291,292].

Supportive and palliative care will remain essential despite progress in precision oncology. Patients with pancreatic cancer frequently experience cachexia, pain, biliary obstruction, pancreatic exocrine insufficiency, venous thromboembolism, and psychological distress [5,231,293]. Early integration of nutritional support, pancreatic enzyme replacement, endoscopic interventions, pain control, optimized celiac plexus block techniques, and palliative care improves symptom control and quality of life [293,294,295,296]. Future precision oncology frameworks should therefore incorporate not only genomic therapeutics but also individualized supportive care based on frailty, functional status, symptom burden, and patient-reported outcomes [293,294,295,296].

Health-economic considerations will become increasingly important as molecular testing, targeted therapies, immunotherapies, and ADCs expand in PDAC [297,298,299,300]. Although precision therapies may provide meaningful benefit in selected patients, their high costs, low biomarker prevalence, and unequal access to genomic testing may limit implementation [297,298,299,300,301]. Future models should integrate real-world evidence, biomarker prevalence, treatment sequencing, toxicity, hospitalization burden, quality-adjusted survival, and disparities in access to specialized care [297,298,299,300,301].

Artificial intelligence, machine learning, digital pathology, and multi-omic profiling may further support early diagnosis, biomarker interpretation, treatment selection, resistance prediction, and clinical trial stratification [302,303,304,305]. Integration of genomic, transcriptomic, proteomic, metabolomic, spatial immune, microbiome, and radiomic data could enable more refined biological subclassification and adaptive therapeutic sequencing. Future translational research may additionally incorporate cancer neuroscience, neural–tumor signaling interactions, and neuroimmune modulation as emerging components of pancreatic cancer biology and therapeutic resistance [286,287,302,303,304,305].

Overall, the future of pancreatic cancer management will depend on multidisciplinary precision oncology frameworks that combine comprehensive molecular profiling, biomarker-guided treatment selection, adaptive systemic therapy sequencing, optimized local therapy, supportive care, and health-economic sustainability. Although major challenges remain, advances in KRAS inhibition, PARP inhibitor maintenance, HER2-directed ADCs, MSI-H/dMMR-directed immunotherapy, and fusion-directed treatment suggest that PDAC management is gradually moving toward more personalized and biologically informed strategies [23,32,33,125,197,267]. Continued progress will require prospective validation of emerging targets, improved biomarker standardization, a deeper understanding of resistance biology, broader access to genomic testing, equitable implementation of precision therapeutics, and continued development of organoid-guided therapeutic testing, advanced biologic delivery systems, tumor microenvironment modulation, CRISPR-based genome engineering, and adaptive immunotherapeutic platforms [241,252,264,306,307].

**Figure 5:**
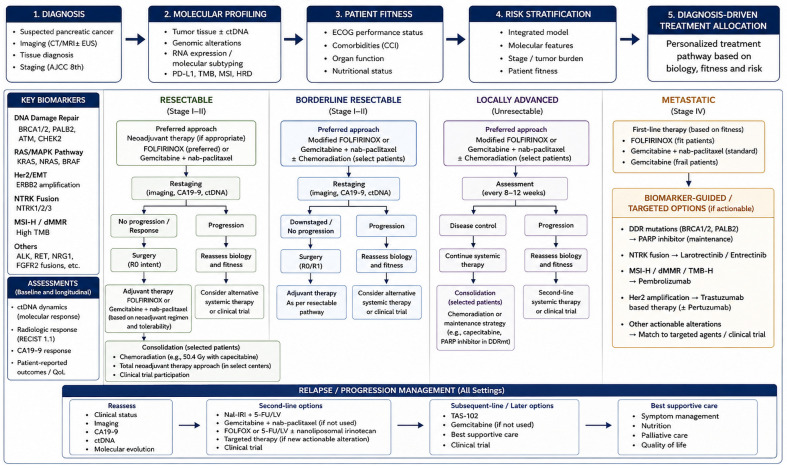
Therapeutic pathways and treatment algorithms in pancreatic cancer integrating biomarkers and treatment sequencing. The figure depicts diagnosis-driven treatment allocation informed by molecular profiling, patient fitness, and risk stratification, integrating targeted therapies, consolidation approaches, and relapse management, following the principal recommendations of NCCN and ESMO guidelines. The proposed treatment algorithm is intended as a literature-based conceptual overview summarizing current evidence from published clinical trials and international guidelines. It is not intended to replace individualized clinical decision-making or multidisciplinary evaluation, which should always consider patient performance status, comorbidities, treatment-related toxicities, molecular findings, physician judgment, and institutional expertise. The scheme does not constitute a direct reproduction of any published guideline, figure, or external source. Selected therapeutic pathways, particularly emerging targeted combinations and maintenance strategies, reflect evolving clinical evidence, contemporary translational insights, and investigational treatment approaches currently under active evaluation rather than prescriptive clinical management recommendations. All abbreviations employed are defined in the Abbreviations section.

Table 7 summarizes contemporary management of pancreatic cancer, including indications, representative regimens, molecular biomarkers, toxicity considerations, and supporting evidence, while Figure 5 outlines diagnosis-driven therapeutic pathways and treatment algorithms integrating molecular profiling and treatment sequencing.

## 6. Conclusions

The therapeutic landscape of pancreatic cancer is gradually evolving toward a more individualized and biomarker-driven model of care. Although conventional chemotherapy remains the cornerstone of treatment, recent clinical advances indicate that selected molecular subgroups of pancreatic cancer may derive clinically meaningful benefit from targeted therapies, ICIs, and ADCs. The clinical development of adagrasib, dostarlimab, and trastuzumab deruxtecan illustrates how distinct molecular alterations—including KRAS G12C mutations, MSI-H/dMMR status, and HER2 overexpression—can be therapeutically exploited in carefully selected patients.

These advances also underscore several principles that are likely to shape future pancreatic cancer management. First, comprehensive molecular profiling is becoming increasingly important for identifying actionable genomic alterations and guiding treatment selection. Second, durable clinical benefit will likely require rational combination strategies capable of overcoming stromal-mediated resistance, tumor heterogeneity, and adaptive signaling pathway reactivation. Third, continued progress in circulating tumor DNA analysis, next-generation sequencing, artificial intelligence, and multi-omic profiling may further improve patient stratification, response monitoring, and treatment optimization.

Despite these encouraging developments, important challenges remain. Most actionable molecular alterations occur in relatively small patient subsets, and much of the currently available clinical evidence—particularly for dostarlimab and trastuzumab deruxtecan in pancreatic cancer—is derived from tissue-agnostic basket studies or small pancreatic cancer cohorts. Consequently, many efficacy estimates should be interpreted cautiously until confirmed in larger prospective pancreatic cancer-specific studies. In addition, acquired resistance, the immunosuppressive tumor microenvironment, and treatment-related toxicity continue to limit durable disease control.

In summary, the integration of targeted therapies, immune checkpoint inhibition, and next-generation ADCs represents an important step toward biologically informed management of pancreatic cancer. Although these approaches currently benefit only selected biomarker-defined patient populations, continued advances in molecular diagnostics, biomarker refinement, resistance biology, and translational research are expected to expand precision therapeutic opportunities and further improve individualized treatment strategies for this highly lethal malignancy.

## Figures and Tables

**Table 1:** TEAEs and management strategies for adagrasib according to [33,75,107,108,109]. All abbreviations employed are defined in the Abbreviations section.

TEAE	Frequency/ Severity	Timing/ Clinical Features	Recommended Management
Diarrhea	Common; mostly grade 1–2, occasional grade ≥ 3	Frequently occurs during the first weeks of therapy; may be associated with dehydration and electrolyte imbalance	Supportive care with hydration, dietary modification, antidiarrheal agents (e.g., loperamide), electrolyte monitoring, dose interruption or reduction for persistent grade ≥ 2 toxicity
Nausea	Very common; predominantly grade 1–2	Early-onset gastrointestinal toxicity; may occur intermittently throughout treatment	Antiemetics, administration with food if tolerated, hydration, dose adjustment for persistent symptoms
Vomiting	Common; mainly grade 1–2	Often accompanies nausea during early treatment cycles	Antiemetics, fluid replacement, electrolyte monitoring, temporary treatment interruption if severe
Fatigue	Common; usually grade 1–2	May develop progressively during continuous therapy	Evaluate contributing factors (anemia, dehydration, endocrine dysfunction), supportive care, activity adjustment, dose modification if clinically significant
Elevated ALT/AST	Common; grade ≥ 3 elevations reported	Typically asymptomatic laboratory abnormality; usually observed during the first 1–3 months	Regular liver function monitoring, temporary interruption for grade ≥ 3 elevations, dose reduction or discontinuation if recurrent or severe hepatotoxicity
Hepatotoxicity	Less common but potentially serious	May present with transaminase elevation, bilirubin increase, or drug-induced liver injury	Close hepatic monitoring, exclusion of alternative etiologies, corticosteroids in immune-mediated overlap cases, permanent discontinuation in severe injury
QT interval prolongation	Uncommon; occasionally grade ≥ 3	May occur at variable timepoints; risk increased with electrolyte abnormalities or concomitant QT-prolonging drugs	Baseline and periodic ECG monitoring, correction of potassium and magnesium abnormalities, avoidance of interacting medications, dose interruption if clinically significant
Anemia	Common; mostly grade 1–2	Gradual onset during prolonged therapy	CBC monitoring, supportive care, transfusion support when indicated, evaluate alternative causes
Decreased appetite	Common; generally low grade	Frequently associated with nausea or fatigue	Nutritional counseling, appetite support measures, hydration, symptom-directed supportive care
Dyspnea	Common; variable severity	May reflect underlying disease progression, pneumonitis, anemia, or cardiopulmonary comorbidity	Clinical assessment including imaging and oxygen saturation, treat underlying cause, interrupt therapy if drug-related toxicity suspected
Peripheral edema	Less common; usually low grade	Can occur during prolonged treatment exposure	Fluid balance monitoring, compression therapy, diuretics when appropriate, dose modification in persistent cases
Renal impairment/increased creatinine	Uncommon; generally reversible	Often associated with dehydration or gastrointestinal toxicity	Renal function monitoring, hydration optimization, correction of electrolyte disturbances, temporary treatment interruption if clinically indicated
Interstitial lung disease/pneumonitis	Rare but potentially life-threatening	May present with cough, dyspnea, hypoxia, or radiographic infiltrates	Immediate interruption of therapy, diagnostic imaging and infectious workup, corticosteroids if suspected drug-related pneumonitis, permanent discontinuation for severe cases
Pancreatitis	Very rare	Abdominal pain with elevated pancreatic enzymes	Treatment interruption, supportive care, pancreatic enzyme monitoring, discontinue permanently in severe cases
Severe hypersensitivity reactions	Very rare	Rash, fever, or systemic allergic manifestations	Immediate discontinuation, antihistamines and corticosteroids as indicated, supportive management
Cardiac arrhythmias	Very rare but clinically significant	Usually associated with QT prolongation or pre-existing cardiac disease	Cardiology evaluation, ECG monitoring, correction of reversible risk factors, discontinue therapy in severe arrhythmias

**Table 2:** Major pivotal clinical trials and selected emerging studies of adagrasib in pancreatic cancer. All abbreviations employed are defined in the Abbreviations section.

Trial/ Study	Population	Cancer Setting	Design	Trial Status	Combination	Key Findings	Inclusion/ Eligibility Criteria
KRYSTAL-1 phase II solid-tumor cohort (NCT03785249) [33]	KRAS G12C-mutated advanced solid tumors excluding NSCLC and CRC; included PDAC	Previously treated advanced/metastatic PDAC	Multicohort, open-label phase I/II basket trial	Completed (mature pancreatic efficacy data available)	Adagrasib 600 mg orally BID	Mature PDAC efficacy results demonstrated an ORR of 33.3% (7/21). In the overall non-NSCLC/non-CRC cohort, ORR was 35.1%, median PFS 7.4 months, and median DOR 5.3 months.	Advanced KRAS G12C-mutated solid tumor; prior systemic therapy; measurable disease; no available standard curative option
KRYSTAL-1 GI-tumor update (NCT03785249) [110]	Pretreated KRAS G12C-mutated PDAC and other non-CRC GI tumors	Unresectable/metastatic gastrointestinal tumors	Phase II exploratory GI cohort	Ongoing (interim pancreatic efficacy data)	Adagrasib monotherapy	Interim PDAC results demonstrated encouraging disease control, supporting further clinical evaluation; mature efficacy outcomes remain pending.	KRAS G12C mutation; unresectable/metastatic PDAC or other GI tumor; prior treatment
MD Anderson phase Ib pancreatic-specific study (NCT05634525) [111]	KRAS G12C-mutated unresectable or metastatic pancreatic cancer	Pancreatic cancer-specific cohort	Phase Ib, single-arm study	Withdrawn	Adagrasib monotherapy	Study was withdrawn before completion; no mature pancreatic efficacy results are available.	Histologically confirmed pancreatic cancer with KRAS G12C mutation; unresectable/metastatic disease; RECIST 1.1 measurable/evaluable disease; ECOG 0–2; no more than one prior therapy
BMS-986466 plus adagrasib study (NCT06024174) [112]	Advanced KRAS G12C-mutated NSCLC, CRC, PDAC, or BTC	Advanced/metastatic KRAS G12C-mutated PDAC included	Phase I/II dose-finding and expansion study	Administratively completed (no pancreatic-specific efficacy data reported)	BMS-986466 plus adagrasib, with or without cetuximab depending on cohort	Designed to evaluate safety, tolerability, and antitumor activity; pancreatic-specific efficacy outcomes have not been reported.	Advanced KRAS G12C-mutated PDAC or other eligible tumor; relapsed/refractory to standard therapy; documented KRAS G12C mutation

**Table 3:** TEAEs and management strategies for dostarlimab according to [125,161,162,163]. All abbreviations employed are defined in the Abbreviations section.

TEAE	Frequency/ Severity	Timing/ Clinical Features	Recommended Management
Fatigue/asthenia	Common; usually grade 1–2	May occur early or accumulate during treatment; nonspecific, often multifactorial	Assess anemia, thyroid dysfunction, infection, nutritional status; supportive care; consider treatment interruption for persistent grade ≥ 3 symptoms
Nausea	Common; mostly grade 1–2, especially with chemotherapy combinations	Early during treatment cycles; may overlap with carboplatin–paclitaxel toxicity	Antiemetics, hydration, dietary modification; manage chemotherapy-related contribution; dose delay if severe
Diarrhea	Common; usually low grade, but may represent immune-mediated colitis	Loose stools, abdominal pain, mucus/blood if colitis develops	Antidiarrheals only after excluding colitis/infection; withhold dostarlimab for grade ≥ 2 immune-mediated colitis; corticosteroids for suspected immune-related toxicity
Rash/pruritus	Common; mostly grade 1–2	Maculopapular rash, itching, or dermatitis; may occur at any time	Topical corticosteroids, antihistamines; dermatology review if persistent; withhold for severe rash; systemic corticosteroids for grade ≥ 3 immune-mediated skin toxicity
Hypothyroidism	Common immune-related endocrinopathy	Fatigue, weight gain, cold intolerance; often detected by abnormal TSH	Monitor TSH/free T4; thyroid hormone replacement; dostarlimab can usually continue if clinically stable
Hyperthyroidism/thyroiditis	Less common; usually low grade	May precede hypothyroidism; palpitations, weight loss, tremor	Monitor thyroid function; beta-blockers for symptoms; endocrinology input; continue if mild, withhold if severe
Pneumonitis	Uncommon but potentially serious/fatal	Cough, dyspnea, hypoxia, new infiltrates; can occur during or after therapy	Withhold dostarlimab; chest imaging and infection workup; corticosteroids for grade ≥ 2; permanently discontinue for grade 3–4 or recurrent severe pneumonitis
Colitis	Uncommon but clinically important	Persistent diarrhea, abdominal pain, fever, blood/mucus in stool	Exclude infection; withhold for grade 2–3; systemic corticosteroids; consider infliximab/vedolizumab if steroid-refractory; permanently discontinue for grade 4
Hepatitis/increased ALT or AST	Uncommon; grade ≥ 3 possible	Often asymptomatic laboratory abnormality; may include bilirubin elevation	Monitor LFTs; withhold for significant elevations; corticosteroids for immune-mediated hepatitis; permanently discontinue for severe or recurrent hepatotoxicity
Adrenal insufficiency/hypophysitis	Rare	Fatigue, hypotension, headache, hyponatremia; may mimic disease-related symptoms	Check cortisol, ACTH, electrolytes; hormone replacement; high-dose corticosteroids if acute adrenal crisis or severe hypophysitis; endocrinology consultation
Nephritis/renal dysfunction	Rare	Rising creatinine, proteinuria, or sterile pyuria	Exclude dehydration, obstruction, nephrotoxins; withhold dostarlimab; corticosteroids for immune-mediated nephritis; nephrology input
Infusion-related reactions	Uncommon; usually grade 1–2	Fever, chills, flushing, dyspnea, hypotension during or shortly after infusion	Interrupt or slow infusion; symptomatic treatment; permanently discontinue for severe or life-threatening reactions
Very rare immune-mediated events	Very rare; may be severe or fatal	Includes myocarditis, encephalitis, meningitis, myasthenia gravis–like syndrome, Guillain–Barré syndrome, pancreatitis, uveitis, severe cutaneous adverse reactions, and solid-organ transplant rejection	Urgent specialist evaluation; hold dostarlimab; high-dose corticosteroids and organ-specific management; permanently discontinue for life-threatening or grade 4 immune-mediated toxicity

**Table 4:** Major pivotal clinical trials and selected emerging studies of dostarlimab in pancreatic cancer. All abbreviations employed are defined in the Abbreviations section.

Trial/ Study	Population	Cancer Setting	Design	Trial Status	Combination	Key Findings	Inclusion/ Eligibility Criteria
GARNET solid-tumor cohort (NCT02715284) [125]	dMMR/MSI-H advanced or recurrent solid tumors, including pancreatic cancer	Biomarker-selected advanced pancreatic cancer subset	Phase I, open-label, multicohort, nonrandomized basket trial	Completed (mature pancreatic efficacy data available)	Dostarlimab monotherapy	Mature pancreatic cancer efficacy data demonstrated an ORR of 41.7% (5/12). In the overall dMMR solid-tumor cohort, ORR was 44.0%, with median DOR not reached.	Advanced/recurrent dMMR or MSI-H solid tumor; prior systemic therapy; measurable disease by RECIST 1.1; no satisfactory standard treatment option
DOVIPA (NCT06757244) [164]	Treatment-naïve metastatic PDAC	First-line metastatic pancreatic cancer	Phase II, open-label, multicenter, nonrandomized study with safety run-in	Ongoing (recruiting; no efficacy data available)	Dostarlimab + mFOLFIRINOX + high-dose oral vitamin D3	Ongoing study designed to evaluate the efficacy and safety of chemoimmunotherapy combined with vitamin D3; pancreatic efficacy results have not yet been reported.	Untreated metastatic PDAC; eligible for mFOLFIRINOX; adequate organ function; measurable disease
Niraparib + dostarlimab study (NCT04493060) [165]	HRR-mutated metastatic PDAC	Previously treated metastatic pancreatic cancer	Phase II, single-arm investigator-initiated trial	Ongoing (no mature efficacy data available)	Niraparib + dostarlimab	Ongoing study evaluating disease control rate, ORR, PFS, OS, safety, ctDNA dynamics, and immune microenvironment changes; mature efficacy results are pending.	Metastatic PDAC with germline or somatic BRCA1, BRCA2, PALB2, BARD1, RAD51C, or RAD51D mutation; ECOG 0–1; 1–2 prior systemic treatment lines; prior platinum therapy unless contraindicated
Niraparib + dostarlimab + radiation therapy (NCT04409002) [166,167]	Metastatic pancreatic adenocarcinoma	Previously treated metastatic pancreatic cancer	Phase II	Closed to accrual (efficacy results pending)	Niraparib + dostarlimab + radiotherapy	Enrollment has been completed; the study is evaluating disease control, PFS, OS, toxicity, cfDNA dynamics, MSI status, and immune responses. Pancreatic efficacy results have not yet been reported.	Metastatic pancreatic adenocarcinoma; ECOG ≤1; ≥1 prior treatment line; measurable lesion suitable for radiotherapy and an additional measurable lesion outside the radiation field
Niraparib + dostarlimab in HRD solid tumors (NCT04983745) [168]	HRD-positive advanced solid tumors, potentially including pancreatic cancer	Biomarker-selected advanced solid tumors	Phase II, open-label, single-arm basket study	Ongoing (no pancreatic-specific efficacy data available)	Niraparib + dostarlimab	Ongoing basket study evaluating combined PARP inhibition and PD-1 blockade in HRD-positive tumors; pancreatic-specific efficacy outcomes have not yet been reported.	Advanced solid tumor with an HRD-associated molecular alteration; measurable disease; adequate organ function

**Table 5:** TEAEs and management strategies for trastuzumab deruxtecan according to [171,172,197,223,224,225]. All abbreviations employed are defined in the Abbreviations section.

TEAE	Frequency/ Severity	Timing/ Clinical Features	Recommended Management
Nausea	Very common; usually grade 1–2, but may require dose modification	Often early after infusion; may be persistent across cycles	Prophylactic and rescue antiemetics, hydration, dietary modification; consider dose interruption/reduction for persistent grade ≥ 2–3 symptoms
Vomiting	Common; mostly grade 1–2	Usually accompanies nausea during early cycles	Antiemetics, fluid/electrolyte replacement; temporary interruption if severe or persistent
Fatigue/asthenia	Common; mostly grade 1–2; grade ≥ 3 reported	May accumulate with continued therapy; may overlap with anemia or disease burden	Evaluate anemia, thyroid dysfunction, infection, nutrition; supportive care, activity adjustment; dose interruption/reduction for grade ≥ 3
Decreased appetite/weight loss	Common; usually low grade	Often associated with nausea, dysgeusia, or fatigue	Nutritional counseling, appetite support, antiemetic optimization; monitor body weight and hydration
Alopecia	Common; usually grade 1–2	Gradual hair thinning or loss during treatment	Patient counseling, scalp care, psychosocial support; no dose modification usually required
Diarrhea	Common; mostly grade 1–2	May occur during early or later cycles; dehydration possible	Antidiarrheal therapy, hydration, electrolyte monitoring; dose interruption if grade ≥ 3 or persistent
Constipation	Common; usually grade 1–2	Often related to antiemetics, reduced intake, or reduced mobility	Hydration, dietary fiber, stool softeners/laxatives; review constipating medications
Neutropenia/decreased neutrophil count	Common; grade ≥ 3 frequent in some cohorts	Laboratory toxicity, usually during early cycles; may increase infection risk	CBC monitoring before each cycle; treatment delay/interruption, dose reduction; G-CSF according to institutional practice and febrile neutropenia risk
Anemia/decreased hemoglobin	Common; grade ≥ 3 reported	Progressive fatigue, dyspnea, pallor; may be laboratory-detected	CBC monitoring, evaluate bleeding/hemolysis/nutritional causes; transfusion support when indicated; dose modification if severe
Thrombocytopenia/decreased platelet count	Common; usually laboratory-detected, grade ≥ 3 possible	Bruising, bleeding, or asymptomatic platelet decline	CBC monitoring; bleeding precautions; dose interruption/reduction for grade ≥ 3; platelet transfusion if clinically indicated
Leukopenia/lymphopenia	Common laboratory abnormality	Usually asymptomatic; may increase infection susceptibility	CBC monitoring; infection surveillance; manage according to severity and associated neutropenia
Increased ALT/AST	Common; usually grade 1–2, grade ≥ 3 possible	Often asymptomatic laboratory abnormality	Baseline and periodic LFT monitoring; exclude hepatic progression or viral/drug causes; dose interruption/reduction for significant elevations
Interstitial lung disease/pneumonitis	Important, uncommon-to-common depending on tumor cohort; can be fatal	Cough, dyspnea, fever, hypoxia, or new radiographic infiltrates; may also be asymptomatic on imaging	Monitor respiratory symptoms and imaging; immediately interrupt T-DXd for suspected ILD/pneumonitis; start corticosteroids for grade ≥ 2; permanently discontinue for grade ≥ 2 confirmed ILD/pneumonitis; consider rechallenge only after resolved grade 1 according to guidance
Infusion-related reactions	Uncommon; usually grade 1–2	Fever, chills, flushing, dyspnea, hypotension during or shortly after infusion	Slow or interrupt infusion; symptomatic treatment with antihistamines/antipyretics; discontinue permanently for severe or life-threatening reaction
Very rare serious events	Very rare, but clinically significant	Includes febrile neutropenia, severe infection, myocarditis, severe hypersensitivity, severe cutaneous reactions, pancreatitis, and fatal ILD/pneumonitis	Urgent specialist evaluation; hold T-DXd; organ-specific treatment; permanently discontinue for life-threatening or recurrent severe toxicity

**Table 6:** Major pivotal clinical trials and selected emerging studies of trastuzumab deruxtecan in pancreatic cancer. All abbreviations employed are defined in the Abbreviations section.

Trial/ Study	Population	Cancer Setting	Design	Trial Status	Combination	Key Findings	Inclusion/ Eligibility Criteria
DESTINY-PanTumor02 (NCT04482309) [197,226,227]	HER2-expressing advanced solid tumors, including pancreatic cancer	Previously treated unresectable/metastatic pancreatic cancer	Phase II, open-label, multicenter, multicohort basket trial	Completed (mature pancreatic efficacy data available)	T-DXd monotherapy	Mature tumor-agnostic efficacy data demonstrated clinically meaningful activity across HER2-expressing solid tumors. The pancreatic cancer cohort was small, showing evidence of antitumor activity, although interpretation was limited by the small sample size.	HER2-expressing advanced solid tumor; prior systemic therapy; no satisfactory standard treatment option; measurable disease; adequate organ function
DESTINY-PanTumor01 (NCT04639219) [228]	Advanced solid tumors with activating HER2 mutations, including pancreatic cancer	Previously treated HER2-mutant advanced/metastatic pancreatic cancer	International phase II, open-label, single-arm basket trial	Ongoing (interim tumor-agnostic efficacy data; pancreatic-specific analysis pending)	T-DXd monotherapy	Interim analyses demonstrated clinically meaningful activity across HER2-mutant solid tumors; pancreatic-specific efficacy data remain limited and have not yet been reported separately.	Activating HER2 mutation; unresectable/metastatic solid tumor; prior standard therapy; measurable disease
Neratinib + T-DXd GI study (NCT05274048) [229]	HER2-overexpressing unresectable/metastatic gastrointestinal cancers, including pancreatic cancer	Advanced HER2-positive pancreatic/GI cancer	Phase I dose-finding study with possible expansion	Ongoing (no pancreatic efficacy data available)	Neratinib + T-DXd	Ongoing study designed to determine the MTD/RP2D, safety, and preliminary antitumor activity of dual HER2 blockade combined with an ADC; pancreatic-specific efficacy results have not yet been reported.	Unresectable/metastatic GI cancer; HER2 overexpression; pancreatic cancer eligible if HER2 IHC 3+; adequate organ function
HER2-positive pancreatic cancer case-level evidence [230]	HER2-positive pancreatic metastasis treated with T-DXd	Pancreatic involvement in HER2-positive disease	Case report	Published case-level evidence (hypothesis-generating)	T-DXd monotherapy	Complete response of a pancreatic metastasis with a reported PFS of 14 months; findings are hypothesis-generating and not representative of clinical trial evidence.	HER2-positive disease with pancreatic metastasis; T-DXd administered following prior HER2-directed therapy

**Table 7:** Contemporary management of pancreatic cancer: clinically focused overview of current treatment strategies, including indications, representative regimens, biomarkers, toxicity considerations, and supporting evidence. The table below integrates contemporary guideline-based and biomarker-driven strategies for pancreatic cancer, including recent updates for metastatic PDAC and actionable molecular subsets. All abbreviations employed are defined in the Abbreviations section.

Modality	Indication/ When Used	Example Regimens/Agents	Key Evidence	Biomarkers/Toxicity
Surgery	Resectable PDAC; selected borderline resectable tumors after response to neoadjuvant therapy	Pancreaticoduodenectomy, distal pancreatectomy, total pancreatectomy	Curative-intent cornerstone in localized disease [231,308]	CA19-9, performance status, vascular involvement; surgical morbidity, pancreatic fistula
Adjuvant chemotherapy	After R0/R1 resection in fit patients	mFOLFIRINOX; gemcitabine + capecitabine; gemcitabine alone if less fit	mFOLFIRINOX improved survival vs. gemcitabine; ESPAC-4 supported gemcitabine + capecitabine [253,254]	ECOG, renal/hepatic function; neutropenia, diarrhea, neuropathy, hand–foot syndrome
Neoadjuvant therapy	Borderline resectable or selected high-risk resectable PDAC	FOLFIRINOX/mFOLFIRINOX; gemcitabine + nab-paclitaxel; chemoradiotherapy in selected cases	Increasingly used to improve margin-negative resection and select biologically favorable disease [309,310]	CA19-9, vascular anatomy, response imaging; myelosuppression, neuropathy, GI toxicity
Chemoradiotherapy/SBRT	Selected locally advanced, borderline resectable, or unresectable non-metastatic PDAC after systemic therapy	Capecitabine- or gemcitabine-based CRT; SBRT	May improve local control in selected patients, although survival benefit is context-dependent [288]	Tumor location, duodenal proximity; enteritis, fatigue, biliary/gastric toxicity
First-line metastatic chemotherapy, fit patients	Metastatic PDAC with ECOG 0–1/selected ECOG 2	FOLFIRINOX; NALIRIFOX; gemcitabine + nab-paclitaxel	FOLFIRINOX and gemcitabine/nab-paclitaxel established standards; NAPOLI-3 supported NALIRIFOX [8,9,256]	ECOG, bilirubin, neuropathy risk; neutropenia, diarrhea, fatigue, neuropathy
First-line metastatic chemotherapy, less fit patients	ECOG 2, frailty, comorbidities, or poor tolerance expected	Gemcitabine ± nab-paclitaxel; gemcitabine monotherapy	Guideline-supported individualized lower-intensity therapy [231,308]	ECOG, age, organ function; cytopenias, fatigue, edema
Second-line therapy	Progression after first-line chemotherapy	Liposomal irinotecan + 5-FU/LV; FOLFOX; FOLFIRI; gemcitabine-based therapy after FOLFIRINOX	NAPOLI-1 supported nanoliposomal irinotecan + 5-FU/LV after gemcitabine-based therapy [311]	Prior therapy, ECOG, bilirubin; diarrhea, neutropenia, fatigue
PARP inhibitor maintenance	Germline BRCA1/2-mutated metastatic PDAC without progression after platinum chemotherapy	Olaparib maintenance	POLO showed improved PFS vs. placebo [260]	Germline BRCA1/2; anemia, fatigue, nausea
Immunotherapy	Rare MSI-H/dMMR or high-TMB PDAC	Pembrolizumab; dostarlimab in dMMR/MSI-H solid tumors	Tissue-agnostic activity in MSI-H/dMMR tumors [41,125]	MSI-H, dMMR, TMB; immune-related colitis, hepatitis, endocrinopathies, pneumonitis
KRAS-targeted therapy	Rare KRAS G12C-mutated PDAC, usually after prior therapy	Adagrasib; sotorasib	KRYSTAL-1 showed activity of adagrasib in KRAS G12C-mutated pancreatic cancer [33]	KRAS G12C; diarrhea, nausea, hepatotoxicity, QT prolongation
HER2-directed therapy	Selected HER2-amplified/overexpressing or HER2-mutant PDAC	Trastuzumab-based combinations; trastuzumab deruxtecan in selected HER2-positive tumors	Basket studies support HER2-directed approaches in selected solid tumors [197]	HER2 IHC/ISH/NGS; ILD/pneumonitis, nausea, myelosuppression
NTRK/NRG1 fusion-directed therapy	Very rare fusion-positive PDAC	Larotrectinib or entrectinib for NTRK fusions; zenocutuzumab for NRG1 fusion-positive PDAC	Tissue-agnostic approvals and NRG1-fusion PDAC data support precision therapy [283,285]	NTRK or NRG1 fusion; dizziness, fatigue, diarrhea, infusion reactions
Supportive and palliative care	All stages, especially advanced disease	Pain control, biliary stenting, pancreatic enzyme replacement, nutrition, thromboembolism management, early palliative care	Essential component of multidisciplinary PDAC care [231,308]	Cachexia, biliary obstruction, pain, VTE; opioid toxicity, stent complications

## Data Availability

No new data were created or analyzed in this study. Data sharing is not applicable to this article.

## Abbreviations

The following abbreviations are used in this manuscript:

5-FU	5-fluorouracil
ADC(s)	antibody–drug conjugate(s)
AE	adverse event
AJCC	American Joint Committee on Cancer
AKT	protein kinase B
ALK	anaplastic lymphoma kinase
ALT	alanine aminotransferase
AST	aspartate aminotransferase
ATM	ataxia telangiectasia mutated
BID	twice daily
BRAF	B-Raf proto-oncogene, serine/threonine kinase
BRCA	breast cancer susceptibility gene
BRCA1/2	breast cancer gene 1/2
BTC	biliary tract cancer
CA19-9	carbohydrate antigen 19-9
CBC	complete blood count
CCI	Charlson Comorbidity Index
CD28	cluster of differentiation 28
CD86	cluster of differentiation 86
cfDNA	circulating cell-free DNA
CI	confidence interval
CRC	colorectal cancer
CRT	chemoradiotherapy
ctDNA	circulating tumor DNA
dMMR	deficient mismatch repair/mismatch repair deficiency
DNA	deoxyribonucleic acid
DDR	DNA damage repair
DOR	duration of response
DXd	deruxtecan payload
ECG	electrocardiogram
ECOG	Eastern Cooperative Oncology Group
EGFR	epidermal growth factor receptor
EMT	epithelial–mesenchymal transition
ERBB2	Erb-B2 receptor tyrosine kinase 2
ERK	extracellular signal-regulated kinase
EU	European Union
EUS	endoscopic ultrasound
FGFR2	fibroblast growth factor receptor 2
FOLFIRI	folinic acid, fluorouracil, and irinotecan
FOLFIRINOX/mFOLFIRINOX	folinic acid, fluorouracil, irinotecan, and oxaliplatin/modified FOLFIRINOX
FOLFOX	folinic acid, fluorouracil, and oxaliplatin
GAP	GTPase-activating protein
G-CSF	granulocyte colony-stimulating factor
GEF	guanine nucleotide exchange factor
GDP	guanosine diphosphate
GI	gastrointestinal
GTP	guanosine triphosphate
HER2	human epidermal growth factor receptor 2
HRD	homologous recombination deficiency
HRR	homologous recombination repair
ICI(s)	immune checkpoint inhibitor(s)
IHC	immunohistochemistry
ILD	interstitial lung disease
iRECIST	immune Response Evaluation Criteria in Solid Tumors
ISH	in situ hybridization
KRAS	Kirsten rat sarcoma viral oncogene homolog
LFTs	liver function tests
LV	leucovorin
MAPK	mitogen-activated protein kinase
MEK	mitogen-activated protein kinase kinase
MHC-II	major histocompatibility complex class II
MRI	magnetic resonance imaging
MSI(-H)	microsatellite instability(-high)
(m)TMB	(measured) tumor mutational burden
MTD	maximum tolerated dose
mTOR	mechanistic target of rapamycin
Nab-paclitaxel	nanoparticle albumin-bound paclitaxel
NALIRIFOX	liposomal irinotecan, fluorouracil, leucovorin, and oxaliplatin
Nal-IRI	nanoliposomal irinotecan
NF-κB	nuclear factor kappa B
NGS	next-generation sequencing
NRAS	neuroblastoma RAS viral oncogene homolog
NRG1	neuregulin 1
NSCLC	non-small-cell lung cancer
NTRK1/2/3	neurotrophic tyrosine receptor kinase 1/2/3
ORR	objective response rate
OS	overall survival
PALB2	partner and localizer of BRCA2
PARP	poly(ADP-ribose) polymerase
PD-1	programmed cell death protein 1
PD-L1	programmed death-ligand 1
PDAC	pancreatic ductal adenocarcinoma
PFS	progression-free survival
PI3K	phosphoinositide 3-kinase
QoL	quality of life
RAL	RAS-like proto-oncogene
RAF	rapidly accelerated fibrosarcoma kinase
RAS	rat sarcoma viral oncogene homolog
R0	microscopically margin-negative resection
R1	microscopically margin-positive resection
RECIST (1.1)	Response Evaluation Criteria in Solid Tumors (version 1.1)
RET	rearranged during transfection
RNA	ribonucleic acid
RP2D	recommended phase II dose
RTK	receptor tyrosine kinase
SBRT	stereotactic body radiotherapy
SHP2	Src homology region 2-containing protein tyrosine phosphatase 2
SOS	son of sevenless homolog
TAS-102	trifluridine/tipiracil
TCR	T-cell receptor
T-DXd	trastuzumab deruxtecan
TEAE	treatment-emergent adverse event
TSH	thyroid-stimulating hormone
US	United States
VTE	venous thromboembolism
